# Intraspecific protoplast fusion of *Brettanomyces anomalus* for improved production of an extracellular β-glucosidase

**DOI:** 10.1080/13102818.2014.955290

**Published:** 2014-11-03

**Authors:** Peng Wu, Xihong Zhao, Siyi Pan

**Affiliations:** ^a^ Hubei Key Laboratory of Economic Forest Germplasm Improvement and Resources Comprehensive Utilization, College of Life Sciences, Huanggang Normal University, Huanggang, Hubei, P. R. China; ^b^ Key Laboratory for Green Chemical Process of Ministry of Education, School of Chemical Engineering and Pharmacy, Wuhan Institute of Technology, Wuhan, Hubei, P. R. China; ^c^ Key Laboratory of Environment Correlative Dietology, Ministry of Education, College of Food Science and Technology, Huazhong Agricultural University, Wuhan, Hubei, P. R. China

**Keywords:** β-glucosidase, *Brettanomyces anomalus* PSY-001, intraspecific protoplast fusion, genome shuffling

## Abstract

Improvement of production of an extracellular β-glucosidase with high activity by *Brettanomyces anomalus* PSY-001 was performed by using recursive protoplast fusion in a genome-shuffling format. The initial population was generated by ultraviolet irradiation, ultrasonic mutagenesis and, then, subjected to recursive protoplast fusion. Mutant strains exhibiting significantly higher β-glucosidase activities in liquid media were isolated. The best mutant strain showed increased cell growth in a flask culture, as well as increased β-glucosidase production. A recombinant strain, F3-25, was obtained after three rounds of genome shuffling and its production of β-glucosidase activity reached 4790 U L^−1^, which was a nearly eightfold increase compared to the original strain *B. anomalus* PSY-001. The subculture experiments indicated that F3-25 was genetically stable.

## Introduction

*Brettanomyces* spp. are described as the non-spore forming counterparts of *Dekkera* spp. and the names of the two genera are often used interchangeably.[1] As *Brettanomyces* is the more common name, it will be used in this paper. The genus *Brettanomyces/Dekkera* includes five species: *Brettanomyces custersianus*, *B. naardenensis*, *B. nanus*, *Dekkera anomala* and *D. bruxellensis*.[2] *Brettanomyces* spp. are found during the fermentation and refermentation of special Belgian beers and ales and are involved in the bioflavouring of a particular Belgian trappist beer.[3,4] The release of flavour-active compounds from hop glycosides opens perspectives for the bioflavouring and product diversification of beverages like beer. Higher activities of exo-β-glucanase can be found in *Brettanomyces* spp. with β-glucosidase activity.[3]

Beta-glucosidases (EC 3.2.1.21) are a group of wide-spread hydrolyases found in microorganisms, plant and animal tissues. Since most of the β-glucosidases from microbial sources are subjected to glucose inhibition, glucose-tolerant β-glucosidases are in high demand.[5] The major applications of β-glucosidases include production of glucose and other soluble sugars from cellulosic wastes in the process of biomass conversion,[6] enhancement of aroma in wine and must by hydrolysis of glycoconjugated precursors [7] and for the enrichment of bioactive isoflavones in soymilk fermentation.[8]

Protoplast fusion is a traditional method, which – although established for over 30 years now – continues to be recognized as a powerful tool for the improvement of many microorganisms. Protoplast fusion shows very broad applicability among microorganisms, both in its intraspecies and interspecies variant, and also between microbes from different kingdoms.[9,10] The technique of protoplast-based genome shuffling has proved to be effective in the rapid improvement of some industrially important microbial strains.[11] Genome shuffling does not require genome sequencing; it is often a preferred method of choice when simultaneous changes have to be introduced at different positions throughout the entire genome. This makes genome shuffling a practical method for the rapid manipulation of complex phenotypes from whole cells and organisms.[12,13]

The aim of this work was to generate high-yield β-glucosidase-producing strains from *B. anomalus* PSY-001 through the combination of protoplast mutation, fusion and genome-shuffling techniques. The mutant strains were subjected to ultraviolet (UV) irradiation, ultrasonic mutagenesis and then, used for recursive protoplast fusion.

## Materials and methods

### Yeast strains

The yeast strain used as the parent strain in this study was *B. anomalus* PSY-001. The identified strain *B. anomalus* PSY-001 was deposited at the China Center for Type Culture Collection (CCTCC) and was given the CCTCC accession number M209106. The strain was stored in Yeast Extract–Peptone–Dextrose (YPD) medium with 20% (v/v) glycerol at –80 °C.

### Medium and cultivation conditions

Yeast strains were grown on 2% YPD medium (1% yeast extract, 2% polypeptone, 2% glucose, and, if necessary, 2% agar) at 30 °C. For the fermentation test of strains, 15% YPD medium (1% yeast extract, 2% polypeptone and 15% glucose) was used. The auxotrophic phenotypes of mutants obtained were determined using minimal medium (MM; 0.17% yeast nitrogen base without amino acids, 2% glucose and 2% agar). If necessary, uracil, L-leucine, L-histidine or L-tryptophan was added to the MM medium to a final concentration of 20, 30, 20 or 20 μg mL^−1^, respectively.

### Strain mutagenesis and mutant screening

Two approaches were employed for mutagenizing the parent strain *B. anomalus* PSY-001. The cells of *B. anomalus* PSY-001 were grown in a shake tube containing a 10 mL of YPD medium at 30 °C for 24 h. First, UV irradiation was performed as follows: 0.2 mL of culture broth was spread onto solid YPD plates and exposed directly to a UV lamp with a wavelength of 254 nm and power of 15 W at a distance of 20 cm for 1–60 s. Second, for ultrasonic mutagenesis, 4 mL of the single-cell suspension was treated with 200 W for 0–55 min. The cells of 0.2 mL culture broth were spread onto solid YPD at the centre of the plate; the lawn around the inhibition zone was scrapped after 48 h incubation, and cultivated in liquid YPD for 12 h. The colonies with larger transparent circles were selected to carry out shake-flask analysis. The strains with higher β-glucosidase activities were obtained and taken as the starter for genome shuffling.

### Protoplast formation

Cell walls were digested using snailase from Yuanye Bio-Technology Co. Ltd (Shanghai, China). Enzymolysis was performed by incubation with 20 mg mL^−1^ snailase for 1 h at 30 °C.

### Protoplast fusion

Biparental-inactivated protoplasts were employed for the protoplast fusion. Protoplast inactivation by UV irradiation was carried out by transferring 5 mL of protoplast suspension to a sterile plate without cover and exposing it for 40 s under a 15 W UV lamp at a distance of 30 cm. Protoplast inactivation by ultrasonic mutagenesis was carried out by transferring 5 mL of protoplast suspension to a sterile plate without cover and exposing it to 200 W ultrasonic treatment for 40 min. The cells of the initial strains subjected to UV irradiation and ultrasonic mutagenesis were inoculated in 250 mL Erlenmeyer flasks containing 20 mL of YPD medium. Cells were harvested at OD660 of about 1.0 by centrifugation at 3500 × *g* for 15 min and were then washed twice with citric acid–sodium citrate buffer.

For protoplast fusion, 0.5 mL of the protoplast suspension from the target strain was mixed and centrifuged for 10 min at 3500 × *g*. The pellet was gently resuspended in 1 mL PE buffer (Yeast Protein Extraction Reagent, Takara) and mixed with 4 mL (40% w/v) polyethylene glycol (PEG) 4000 in PE buffer. After 5 min incubation at 30 °C, the mixture was collected and washed three times with 5 mL of PE buffer (by centrifugation for 5 min at 2000 × *g*). The collected pellet was resuspended in 5 mL of PE buffer and diluted with the same PE buffer at a 1:3 ratio. This diluted suspension (0.1 mL) was plated on regeneration medium and incubated at 30 °C for 3 days.

### Genome shuffling of five high-yield β-glucosidase-producing strains

For genome shuffling, 0.5 mL aliquots of the protoplast suspension of each strain were mixed together. After 10 min centrifugation at 500 × *g*, the pellet was gently resuspended in 1 mL of PE buffer and then mixed with 4 mL (40% w/v) PEG 4000 in PE buffer and incubated at 30 °C for 10 min. The resulting mixture was washed with PE buffer and diluted as described above. The diluted suspension (0.L mL) was plated on regeneration medium and incubated at 30 °C for 3 days.

### Bioassay and shake-flask screening for mutants

A high-throughput cultivation method was employed to rapidly screen large numbers of resulting mutants as described earlier.[14] The fermentation medium in the shake flask was composed of bran (53.15 g L^−1^), yeast extract powder (3.03 g L^−1^), KCl (0.204 g L^−1^) and CaCl_2_ (0.611 g L^−1^); the pH was adjusted to 6.8 before sterilization. This culture medium has been optimized to support efficient production of β-glucosidase with *B. anomalus* in our laboratory and used throughout all the shake-flask experiments.

### Enzyme assay

The β-glucosidase activity was determined with *p*-nitrophenyl-β-D-glucopyranoside (*p*-NPG, Sigma, USA) as a substrate, hence called pNPGase activity, as follows: 1 mL of 5 mmol L^−1^ p-NPG (in 50 mmol L^−1^ citric acid–sodium citrate buffer pH 4.8) was incubated with the enzyme solution at 50 °C for 10 min. The reaction was stopped by adding 1 mL of sodium carbonate (1.0 mol L^−1^, pH 10); the liberated *p*-nitrophenol was measured at 405 nm. Then, one unit of enzyme activity was determined as the amount of enzyme required to release 1 μmol of *p*-nitrophenol in 1 min under the assay conditions.[15]

## Results and discussion

### Strain mutagenesis and mutant screening

**Figure 1.**
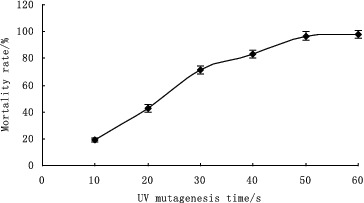
Mortality rate of *B. anomalus* PSY-001 cells exposed to UV with different duration of treatment.

**Figure 2.**
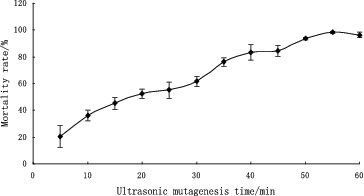
Mortality rate of *B. anomalus* PSY-001 at different duration of ultrasound treatment.

**Figure 3.**
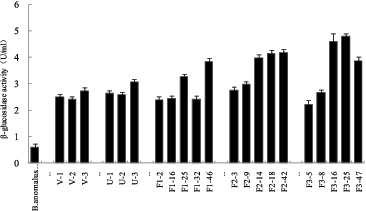
β-glucosidase activity of *B. anomalus* strains.

Genome shuffling practically mimics the features of natural evolution through recursive genetic recombination. Thus, it requires a diverse population of mutants with an improvement of the desired phenotype compared with the wild type as the starting point.[16] In our study, UV and ultrasonic treatment were used as mutagenizing agents to improve the volumetric productivity of the wild-type strain. Figures 1 and 2 show the mortality rate of *B. anomalus* PSY-001 cells after treatment with UV light and ultrasound, respectively. As shown in the figures, cells of the wild-type strain were found to be sensitive to UV irradiation and ultrasound. The mortality rate of the cells increased the longer the exposure to the mutagenizing agents was. Cell suspensions with about 80% mortality rate, which had been exposed to UV light irradiation for 40 s or to ultrasonic treatment for 40 min, were used in the subsequent strain screening. Six mutants, V-1, V-2, V-3, U-1, U-2 and U-3, were obtained by the first series of experiments with UV irradiation and ultrasonic treatment from 100 colonies. These mutants showed a large increase in β-glucosidase enzyme activity from 598 to 3072 U L^−1^ in comparison with *B. anomalus* PSY-001 (Figure 3).

### Genome shuffling of improved mutant population

The efficiency of genome shuffling is known to be a function of the efficiencies of generation and fusion of protoplasts, of recovery of protoplasts after fusion.[17] In our experiments, protoplasts were generated by treatment with snailase to digest the yeast cell wall. Initial experiments to optimize conditions for formation and regeneration of protoplasts were carried out with the three strains of *B. anomalus* PSY-001 that were to be used for protoplast fusion experiments.

Three successive rounds of protoplast fusion were carried out, and after each round, the pH of the plates used for selection was decreased. Screening was done at the end of each fusion and colonies having both traits selected for the next round. After the first fusion, the best five colonies (F1-2, F1-16, F1-25, F1-32 and F1-46) were selected from the first shuffled library and used for the second fusion. Five colonies (F2-3, F2-9, F2-14, F2-18 and F2-42) were obtained from the second shuffled library and used for the next fusion. After the third fusion, five colonies (F3-5, F3-8, F3-16, F3-25 and F3-47) were obtained from the third shuffled library. The results are shown in Figure 3.

Among the five strains, two selected isolates (F3-16 and F3-25) showed β-glucosidase activities of 4610 and 4790 U L^−1^, respectively, which were nearly eightfold higher as compared to *B. anomalus* PSY-001, and were selected for the subsequent studies.

The methods commonly used for improvement of industrial microorganisms range from classical strain improvement techniques to metabolic engineering. Although classical strain improvement is effective, it is very time- and labour-consuming. Protoplast-related techniques have extensive application in the fermentation industry as well as in genetic engineering and molecular biology. These techniques can provide stable changes in the genome of *Brettanomyces* spp. In the present study, genome shuffling was successfully applied as an effective whole-cell engineering strategy for the rapid improvement of industrially important microbial phenotypes. Our results support the assertion that genome shuffling offers more advantages than classical strain improvement strategies and rational genetic methods for strain improvement (i.e. metabolic engineering).[18]

### Genetic stability of F3-25

To check the genetic stability of F3-25, we cultured the shuffled mutant from three successive rounds of protoplast fusion for eight generations and measured the β-glucosidase activities of every other generation. All the generations showed similar activity as the initial strain, suggesting that F3-25 was genetically stable and suitable for the next steps of investigation and industrial production. This makes the strain promising for use in food flavour production.

## Conclusions

In the present work, a recombinant strain, F3-25, was obtained after three rounds of genome shuffling and its production of β-glucosidase activity reached 4790 U L^−1^, which was a nearly eightfold increase compared to the original strain *B. anomalus* PSY-001.
